# Beyond Binaries: Dissolving the Empirical/Normative Divide

**DOI:** 10.1080/23294515.2020.1722290

**Published:** 2020-02-25

**Authors:** Sarah Chan, Sarah Cunningham-Burley, Giulia de Togni, Sonia Erikainen, Nayha Sethi, Kayo Takashima

**Affiliations:** a Centre for Biomedicine, Self and Society, University of Edinburgh, Edinburgh, UK;; b Center for iPS Cell Research and Application, Uehiro Research Division for iPS Cell Ethics, Kyoto University, Kyoto, Japan

In bioethics, the contrast between normative and empirical approaches has commonly been aligned with disciplinary differences: bioethics defines itself as a form of normative inquiry, while empirical methods have traditionally been viewed as principally the tools of the social sciences (Chan and Coggon 2013). Framed in this way, the empirical bioethics enterprise appears as a form of “disciplinary sociality”: what can social science do for bioethics, we might ask; or, perhaps more equitably, what can bioethics and social science do together?

The very fact of asking questions about the relationship between bioethics and social science, however, itself suggests a separation between the two, which in turn predisposes toward binary thinking about the normative and the empirical. Such a binary is subject to continuous re-negotiation: the “empirical turn” in bioethics has been paralleled to some extent by a “normative turn” in the social sciences, bringing us to a place where disciplinarity, methodology, and epistemology intertwine and are both elided and challenged. From a bioethical perspective, this prompts us to ask whether empirical bioethics involves importing social science epistemologies into bioethical work, or is merely the application of empirical methods in a bioethical cause. Empirical bioethics methodologies are notably diverse; their theoretical approaches equally if not more so (Ives et al. 2018); yet what might be missed, if we import empirical methods without also adopting a social scientific approach to theory and epistemology? Questions about epistemic priority – whether empirical evidence serves as justification for normative claims, or whether we start from an agnostic position with respect to normativity and allow the empirical work to drive our analysis – likewise tend to emphasize the idea of difference, rather than alignment.

As a group of scholars striving to move from multi-disciplinarity to interdisciplinarity (Pickersgill et al. 2018; 2019), our own conversations about discipline are often framed by both the expertise we are willing to claim and our acknowledgment of its limits. Disciplinary boundaries are seen to align with, and to define “what I can do [as an academic] versus what I can’t.” Interrogating the differences and relationships between disciplines tends to invoke discourses of epistemic authority: the process of delineating and defining disciplinary identities involves a kind of territoriality, legitimizing certain sorts of knowledge claims as “native” to a given discipline while marking others out as “foreign.” When thinking about the relationship between empirical and normative approaches, we need also to recognize the implications of this as a form of boundary work (see Gieryn 1983), and to reflect on its (perceived) necessity as well as its possible repercussions. What purposes does this boundary work serve? What conversations might be shut down (or opened up) by this, and what barriers to collaboration does it present?

In some ways, the empirical/normative distinction is itself an artifact of bioethical responses to challenges regarding disciplinary identity and purpose. The development of bioethics as a field has been shaped by its emergence at the interface of multiple professions and disciplines, with different objectives, varying approaches to knowledge production, and competing claims to expertise – a process now being recapitulated in the present consideration of the sub-field of empirical bioethics. Attempts to forge a common identity from these disparate elements have produced an account of bioethics as possibly pluralistic with respect to discipline, but united in being problem-oriented and “action-guiding” (Sheehan and Dunn 2013; Ives 2014). Through this discourse, a normative orientation has become a key element in our understanding of what bioethics is and does, and, relatedly, how we legitimise our identity as bioethicists. From this perspective, the empirical is often constructed via its difference from the normative: as descriptive, concerned with the “is” rather than the “ought” (Chan and Coggon 2013).

Attempting to examine this distinction from (at least some) social science perspectives, however, yields a much less clear-cut picture. Many forms of what bioethicists might consider to be “empirical approaches,” associated with social science, both embed and critically acknowledge normativity. Indeed, in our own work we have observed that the use of the term “normative” itself may also be contested across disciplines. Where bioethics understands “normative” to be making a claim about how the world ought to be, regardless of how it is, a social scientist may use the word “normative” to indicate something about how the world is while *simultaneously* acknowledging the way in which this inevitably shapes societal conceptions of the “ought.” In this way, social science perspectives offer potential to go beyond the related “is/ought” and ‘“act/value” distinctions via critiquing their implicit ontological presumptions – that something simply “is,” or that facts have independent existence.

To be sure, some social science approaches take a more positivist stance with respect to “social facts” and some are less willing to engage with normativity. In problematizing the normative/empirical distinction and how it has assumed salience in bioethics, it is important to recognize that parallel boundary work has also been carried out from within the social sciences; certain schools of hardline social empiricism disavow making any claims about “how things ought to be” as illegitimate to their disciplinary orientation, staking their territory as purely to report “how things are.” Digging disciplinary ditches, however, where the normative and the empirical are allocated to different disciplines, reinforces old and hegemonic approaches to knowledge production, which in turn may hamper efforts at collaboration.

More generally, the sort of boundary work that emphasizes and reifies disciplinary differences often comes about as a response to the common challenges faced by scholarship that attempts to transcend discipline or defies (simple) disciplinary classification. Possibly inevitable amongst these, given the extent to which discipline is linked to methodology and epistemology, is the problem of demonstrating robustness when taking hybrid approaches. “Traditional” bioethics often assumes its methodology to be *sui generis*, without the need for explanation, whereas social science centers its methodologies explicitly as crucial to the validity of knowledge. This poses a problem for how to generate knowledge that counts and is respected as valid when working at the interface between disciplines. From an empirical bioethics standpoint, Ives (2014) has suggested a “reflexive bioethics” methodology. Moving beyond bioethics as a disciplinary identity, this approach nonetheless marries well with social science’s general critical reflexivity with respect to methodology, perhaps providing some stability underfoot for researchers venturing further into interdisciplinary territory.

Interdisciplinary (and inter-epistemic) work also faces related issues with respect to its audience and finding the appropriate forum for publication; reviewer comments along the lines of “but it’s not bioethics” will no doubt be familiar to many. This demands of us as researchers that we push boundaries: our own in terms of where we seek audiences for our work, and those of our audiences, when we bring them work that may challenge disciplinary expectations and conventions. It requires us to be aware of when we are falling back on disciplinary boundary work and to maintain an attitude of critical reflexivity as to whether it is warranted, with skepticism being the default position.

Entangled with these issues are a host of others, many related to disciplinary authority and legitimacy: for example, different ideas regarding what constitutes quality and achievement in research. Interdisciplinarity is difficult to achieve on one’s own, at least at the outset; focusing on sole-authored papers as a mark of achievement, as some disciplines have tended to do, undervalues collaboration. Likewise, many productive interdisciplinary interactions occur in less traditionally-scholarly spaces, such as in the context of public engagement or creative activity. An openness to recognizing quality and contribution in a wider variety of forms can reward and encourage, instead of penalize, interdisciplinarity. We also need collegial relationships that enable interdisciplinary critiques. For example, it has been suggested that incorporating social sciences can provide an internal critique of bioethics itself (Haimes 2002), but this requires appropriate humility and respect, trust and confidence, to listen and respond, rather than relapse into more familiar boundary discourse.

Returning, then, to the empirical/normative relationship and its place in interdisciplinary bioethics, one way to go beyond binaries and begin to address these issues differently may be to adopt a feminist epistemology, that actively resists disciplinary divisions and the empirical/normative distinction as necessary to making sense of the world. Feminist scholarship has long theorized ontology and epistemology and the empirical and the normative as co-constitutive, intertwined in inseparable ways (see for example Alcoff and Potter 1993); this has resulted in significant contributions to the theory and methodology of empirical bioethics (Scully 2017). Taking such an approach without having to declare it as either bioethics or social science can assist us to develop and express a reflexive orientation, to iteratively problematize the questions we ask as well as the distinctions we surface or create.

This may mean putting the boundary work to one side: worrying less about what we call ourselves, allowing ourselves to be vulnerable with respect to the new knowledges we might produce, and maintaining openness as well as reflexivity in our approaches to generating them. As scholars, we are playing in a range of fields; we should overcome our preoccupation with the fences we might build (or imagine) between them, and devote more attention to what, in this fertile terrain, we might grow together.

## Author contributions

All authors were involved in the conception, design and data generation for the manuscript, using an interactive discussion format to develop the content and themes. SC wrote the primary draft and all authors had input into its revision and approved the final submission.
